# Early introduction of solid feeding and early cessation of breastfeeding

**DOI:** 10.1111/mcn.13049

**Published:** 2020-06-16

**Authors:** Charlotte M. Wright, Angelina Lessa, Ada Garcia, Pauline Emmett, Sarah Crozier, Sian Robinson, Keith M. Godfrey

**Affiliations:** ^1^ Department of Child Health, School of Medicine, Nursing and Dentistry University of Glasgow Glasgow UK; ^2^ Department of Human Nutrition School of Medicine, Nursing and Dentistry University of Glasgow Glasgow UK; ^3^ Centre for Child and Adolescent Health, Population Health Sciences, Bristol Medical School University of Bristol Bristol UK; ^4^ MRC Lifecourse Epidemiology Unit University of Southampton Southampton UK; ^5^ NIHR Southampton Biomedical Research Centre University of Southampton and University Hospital Southampton NHS Foundation Trust Southampton UK

We thank Perkin and colleagues for their interest in our paper and apologise for omitting to mention the Enquiring About Tolerance (EAT) Trial (Perkin et al., 2016). In response to their letter we would highlight that, although the EAT trial randomly allocated infants to receive certain allergenic solids from 3 to 4 months, breastfeeding cessation was not one of the trial's pre‐specified outcomes (http://www.isrctn.com/ISRCTN14254740), and its findings are hard to generalise. To be eligible to participate, mothers had to be still breastfeeding exclusively at age 3 months, and the breastfeeding cessation rates overall were exceptionally low, so that the study would have been underpowered to detect possible differences in breastfeeding cessation. The amounts of food eaten by infants in the trial were very modest; only 40% consumed more than of 15 g of allergenic proteins per week (the minimum amount specified in the protocol), which is the energy equivalent of less than one stage 1 baby jar per day. It is reassuring that the small amount of solid foods taken before 6 months in the EAT trial did not displace breast milk intake. However, this may not inform the real‐world situation, where larger quantities of solid foods are generally given.

Our observational cohorts demonstrate that the commencement of solids and the cessation of breastfeeding are related but, as stated in our paper, we accept that it seems unlikely that the amount of breast milk displaced by solid foods would, alone, be sufficient to cause secondary lactation failure (Lessa et al., 2020). We speculated that the underlying mechanism is via increased use of formula milk. Complementary solids are still often called ‘weaning foods’, and mothers may view the commencement of solids as a time when breast milk can be augmented with formula milk or stopped altogether. This led us to suggest that any reversal of WHO recommendations on age of first solids could lead to more children losing the protective benefits of breastfeeding.

Thus, any instruction to start solids before 6 months needs to have strong public health justification, based on a full review of both the importance of the competing risks and the strength of evidence for them. Food allergy is a major concern for many families. There seems little doubt that introducing allergenic solids when offering first complementary foods is safe and may be beneficial (Scientific Advisory Committee on Nutrition, 2018). However, the large and well‐designed EAT trial did not find any protective benefit from introducing allergenic solids before 6 months. Furthermore, the interim review cited by Perkin and colleagues has been superseded by two more substantial reviews; these both concluded that the evidence base is too weak to justify starting allergenic solids before 6 months (EFSA Panel on Nutrition et al., 2019; Scientific Advisory Committee on Nutrition & Committee on Toxicity, 2017). Current evidence therefore does not support a change to infant feeding guidance to recommend earlier introduction of solid foods.

## CONTRIBUTIONS

CW drafted the response, and the other authors have commented on successive drafts.
